# Identification of a Phage Display-Derived Peptide Interacting with the N-Terminal Region of Factor VII Activating Protease (FSAP) Enables Characterization of Zymogen Activation

**DOI:** 10.1021/acschembio.2c00538

**Published:** 2022-09-07

**Authors:** Sebastian Berge-Seidl, Nis Valentin Nielsen, Armando A. Rodriguez Alfonso, Michael Etscheid, Sai Priya Sarma Kandanur, Bengt Erik Haug, Maria Stensland, Bernd Thiede, Merve Karacan, Nico Preising, Sebastian Wiese, Ludger Ständker, Paul J. Declerck, Geir Åge Løset, Sandip M. Kanse

**Affiliations:** † Oslo University Hospital and Medical Faculty, University of Oslo, 0372 Oslo, Norway; ‡ Ulm University Medical Center, 89081 Ulm, Germany; § Paul Ehrlich Institute, 63225 Langen, Germany; ∥ Department of Chemistry and Center for Pharmacy, University of Bergen, 5007 Bergen, Norway; ⊥ Department of Biosciences, University of Oslo, 0371 Oslo, Norway; # Department of Pharmaceutical and Pharmacological Sciences, Katholieke Universiteit Leuven, 3000 Leuven, Belgium; ∇ Nextera AS, 0349 Oslo, Norway

## Abstract

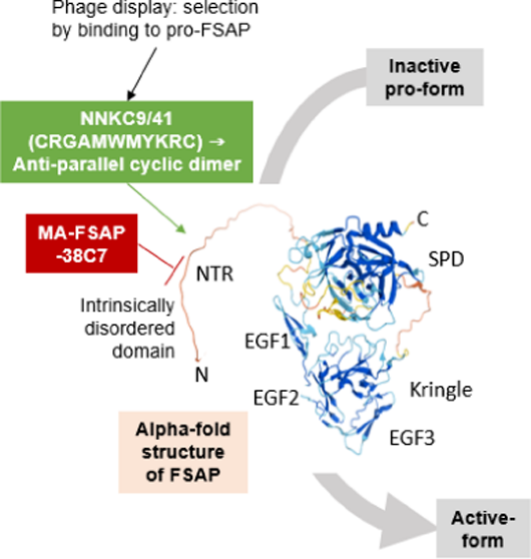

Factor VII Activating protease (FSAP) has a protective effect in diverse disease conditions as inferred from studies in FSAP^–/–^ mice and humans deficient in FSAP activity due to single-nucleotide polymorphism. The zymogen form of FSAP in plasma is activated by extracellular histones that are released during tissue injury or inflammation or by positively charged surfaces. However, it is not clear whether this activation mechanism is specific and amenable to manipulation. Using a phage display approach, we have identified a Cys-constrained 11 amino acid peptide, NNKC9/41, that activates pro-FSAP in plasma. The synthetic linear peptide has a propensity to cyclize through the terminal Cys groups, of which the antiparallel cyclic dimer, but not the monocyclic peptide, is the active component. Other commonly found zymogens in the plasma, related to the hemostasis system, were not activated. Binding studies with FSAP domain deletion mutants indicate that the N-terminus of FSAP is the key interaction site of this peptide. In a monoclonal antibody screen, we identified MA-FSAP-38C7 that prevented the activation of pro-FSAP by the peptide. This antibody bound to the LESLDP sequence (amino acids 30–35) in an intrinsically disordered stretch in the N-terminus of FSAP. The plasma clotting time was shortened by NNKC9/41, and this was reversed by MA-FSAP-38C7, demonstrating the utility of this peptide. Peptide NNKC9/41 will be useful as a tool to delineate the molecular mechanism of activation of pro-FSAP, elucidate its biological role, and provide a starting point for the pharmacological manipulation of FSAP activity.

## Introduction

Factor VII activating protease (FSAP) is a serine protease encoded by the hyaluronic acid binding protein2 (*HABP2*) gene and is predominantly expressed in the liver. A naturally occurring mutation (Gly534Glu) renders the protease inactive,^1^ and this single-nucleotide polymorphism (SNP), termed Marburg I (MI) SNP, is present in 5% of the Caucasian population. It is associated with an enhanced risk of stroke^2^ and carotid stenosis.^3^ In the case of venous thrombosis,^4,5^ the link is contentious.^6,7^ FSAP^–/–^ mice exhibit increased liver fibrosis,^8^ stroke,^9^ and neointima formation,^10^ but the extent of thrombosis is lower.^11^ Hence, a lower FSAP activity is linked to an increased disease burden in both mice and humans. We have demonstrated that the recombinant serine protease domain of FSAP has a positive effect on stroke outcomes in mouse models.^12^ Pro-FSAP circulates in the blood as a single-chain inactive zymogen, and we hypothesize that pharmacologically activating it could also be of therapeutic interest.

Negatively charged polymers such as heparin,^13,14^ nucleic acids,^15,16^ and dextran sulfate^13^ activate purified pro-FSAP but not pro-FSAP in plasma, whereas positively charged histones^17^ and positively charged surfaces^18^ activate pro-FSAP in blood, plasma, and *in vivo.*(17,18) The activation of pro-FSAP is induced by the interaction of anions or cations with the N-terminal region (NTR) or the EGF3 domain of FSAP, respectively. Yamamichi et al. have suggested that charged molecules induce a conformational change, promoting dimerization of pro-FSAP molecules. This leads to cleavage of the interacting partner molecule at the activation site Arg313–Ile314, leading to full activity.^19^

FSAP activity in the circulation increases after surgery and in patients with sepsis,^20^ trauma,^21^ stroke,^22^ and acute respiratory distress syndrome,^23^ which suggests that pro-FSAP activation is related to tissue damage and inflammation. Histones have ubiquitous effects in plasma, blood, and cells,^24,25^ and the activation of FSAP may be part of their function as damage-associated molecular patterns (DAMPs). Active FSAP can cleave and inactivate free histones and limit their cytotoxicity.^17,26,27^ Other substrates of active FSAP include proteins from the hemostasis^28^ and complement system,^21^ growth factors,^29^ and protease-activated receptors (PARs).^30^

Currently, it is not known whether the activation of pro-FSAP is simply dependent on charged macromolecules or whether there is a higher specificity to this process. Phage display has been used as a strategy to identify peptides that activate the zymogen form of proteases.^31^ We hypothesized that a phage display screen could be used to find peptidic modulators of pro-FSAP activity. This approach led to the identification of a peptide that can activate pro-FSAP in a specific, selective, and potent manner. Furthermore, we discovered an inhibitory antibody that blocks pro-FSAP activation by the peptide and demonstrate the efficacy of the peptide and the antibody in plasma clotting assays. Thus, the molecular characterization of the pro-FSAP activation through the development of unique reagents indicates that this is a precise mechanism amenable to pharmacological exploitation.

## Results and Discussion

### Selection of Pro-FSAP Binding Phages

**Figure 1:**
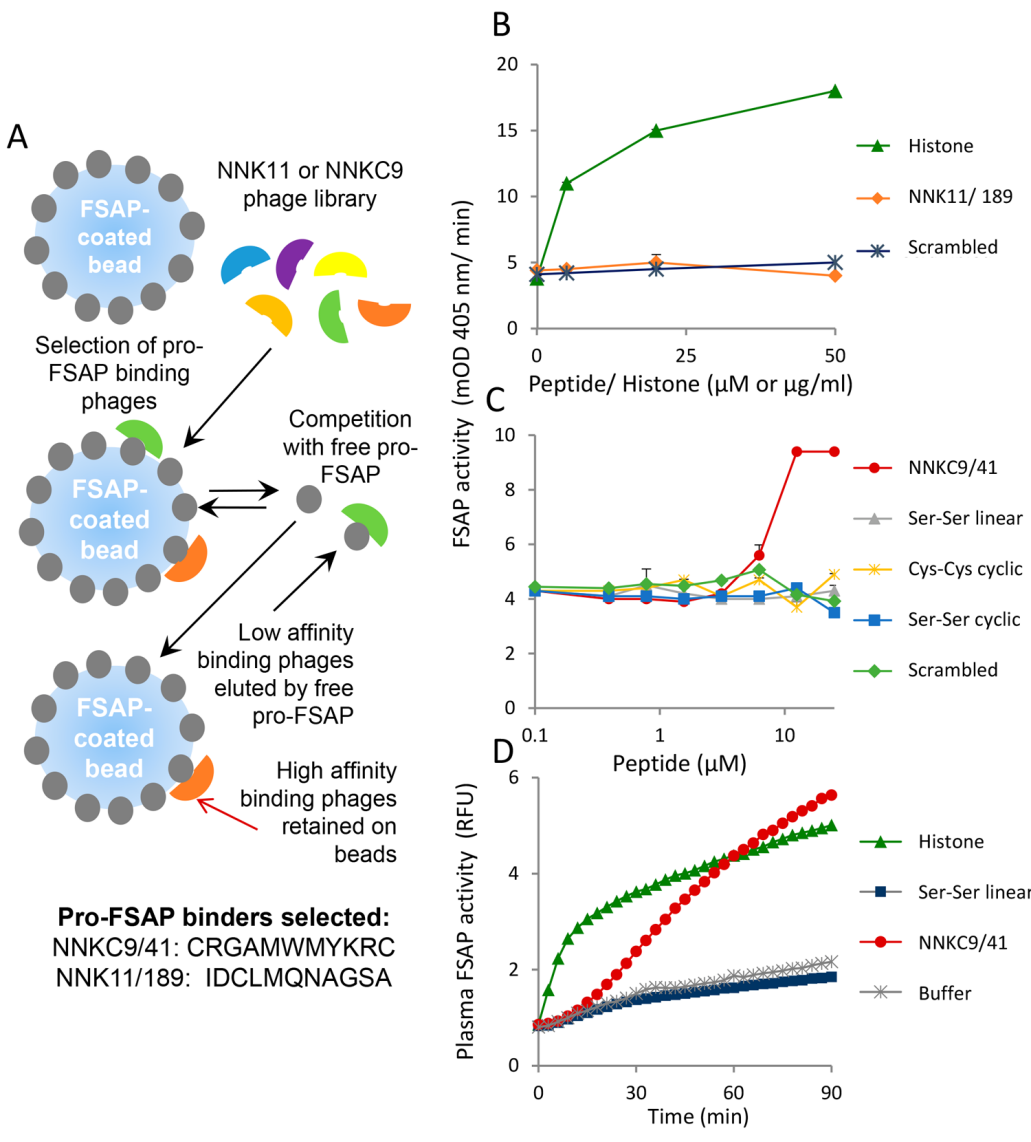
Isolation of pro-FSAP binding peptides and their activity: (A) strategy to isolate high-affinity phages from the NNKC9 and NNK11 libraries that bind to pro-FSAP. To select for strong binders, an excess of free pro-FSAP protein was used for competitive elution of low-affinity binders. High-affinity binders retained on the beads were analyzed further. The sequences of major positive clones identified through an ELISA-based binding assay from the NNKC9 and NNK11 libraries are shown. (B) NNK11/189 and scrambled controls (0–50 μM) and histones (0–50 μg/mL) were added to purified pro-FSAP (1.0 μg/mL) and the turnover of the chromogenic substrate S-2288 (200 μM) was measured. (C) NNKC9/41 was compared to a peptide that was synthetically monocyclized (Cys–Cys cyclic), a peptide with Ser residues instead of Cys at both ends (Ser–Ser linear), a peptide with Ser residues at the end cyclized head to tail (Ser–Ser cyclic), and a scrambled peptide in a pro-FSAP activation assay. (D) Hirudin plasma (1:12 dilution) was incubated with histones (25 μg/mL), NNKC9/41, or Ser–Ser linear control (each, 25 μM), the fluorogenic substrate turnover (Ac–Ala–Lys–Nle–Arg–AMC) was monitored in duplicate wells, and results from a single well are shown. In panels (B) and (C), results are shown as mean + range (duplicate wells). Results were replicated with five different batches of NNKC9/41 (panels (B) and (C)).

To identify peptidic binders and modulators of pro-FSAP activity, we designed a peptide phage display approach to selectively enrich for strong binders using phage selection against biotinylated pro-FSAP purified from human plasma. Two parallel approaches were chosen, where either a random 11-mer (NNK11) or a Cys-constrained random 9-mer (NNKC9) peptide library (formally an 11-mer) was panned in three iterative rounds with a decreasing amount of the bead-immobilized target protein in each round. The selection was tuned to favor binders with high affinity by altering the peptide display levels from high to low valency in later selection rounds and using excess pro-FSAP as a soluble competitor to deplete low-affinity binders in all three rounds (Figure 1A). We then screened phage clones from the third selection round of each library in a phage enzyme-linked immunosorbent assay (ELISA)-based pro-FSAP binding assay. From the NNKC9 library, 5/6 positive binding clones showed the sequence CRGAMWMYKRC, termed NNKC9/41, and the remaining clone had the sequence CEGLAIQVKQC (Figure 1A). From the NNK11 library, 10/11 positive binding clones had the sequence IDCLMQNAGSA, termed NNK11/189, and the remaining clone had the sequence DLPWSMPRPCR (Figure 1A). Thus, we identified two predominant sequences capable of binding to pro-FSAP (sequences in Figure 1A and Supporting Table 1) that were investigated further.

### Activation of Pro-FSAP by NNKC9/41 but Not NNK11/189

To test the effects of these synthetic peptides on FSAP activity, we measured the activation or inhibition of pro-FSAP, purified from human plasma, using a chromogenic substrate (S-2288). The high basal activity of pro-FSAP preparations is due to its propensity to convert to the active form during its purification. Histones were used as a positive activator in all of these experiments. NNK11/189, or its scrambled control (see Supporting Table 1 for sequences), did not have any effect on pro-FSAP activation compared to the strong effect of histones (Figure 1B).

Cys-constrained M13 phage peptide libraries, e.g., NNKC9, primarily display monocyclic peptides as the capsids are assembled into the virions in the oxidized periplasma of *E. coli*.^32^ However, NNKC9/41 synthesized as a monocyclic peptide with an internal disulfide bond showed no effect (Figure 1C). Surprisingly, the same peptide synthesized in a reduced linear form and stored under oxidative conditions was able to robustly activate pro-FSAP (Figure 1C). Maximal activation was 2–2.5-fold, and the EC_50_ was 8–15 μM. We then assessed the importance of the Cys residues by replacing them with Ser residues. A linear peptide with two Ser residues (Ser–Ser linear) and the head-to-tail monocyclized version (Ser–Ser cyclic) failed to activate pro-FSAP (Figure 1C). Scrambling the NNKC9/41 peptide sequence also led to a complete loss of activity (Figure 1C). These results were replicated with five different batches of the synthetic linear peptide and four batches of the monocyclic peptide from two different commercial suppliers, indicating that the activity was stable and reproducible. We reasoned that the activity of the linear Cys-flanked peptide resided not in a cyclic monomer, but in cyclic or linear multimeric forms that could be generated through head-to-head, tail-to-tail, or head-to-tail disulfide bond formation. The formation of noncovalent aggregates was also a possibility.^33^

To identify the nature of the active component, we performed reverse phase-high performance liquid chromatography (RP-HPLC) of linear NNKC9/41 followed by analysis of fractions for pro-FSAP activation as well as liquid chromatography–mass spectrometry (LC–MS). Linear, monocyclic, and cyclic dimers with varying degrees of oxidation were identified in the fractions. The cyclic dimer with varying degrees of oxidation was found in all of the active fractions (Supporting Figure 1). Digestion with LysC, which cleaves at the C-terminal end of Lys, produced a pattern of fragmentation that was compatible with an antiparallel cyclic dimer configuration (Supporting Figure 2).

A purified antiparallel cyclic dimer had a similar activity profile to NNKC9/41 except that the maximal activity was slightly lower, and it caused a pronounced decrease in activity at higher concentrations (Supporting Figure 3). For these studies, we used a fluorescent substrate (Ac–Pro–dTyr–Lys–Arg–AMC)^34^ that was more specific for FSAP. Thus, the cyclic dimer has the appropriate size and spacing between positively charged residues, which bestows it with histone-like properties, but this is not the case for the monocyclic and linear peptides with the same primary sequence. Taken together, the reduced/linear synthetic peptide forms mono- and dimeric-cyclic species under oxidative conditions, of which the latter, in an antiparallel configuration, is the active component. Hereafter, this active mixture of peptides is referred to as NNKC9/41.

The next question was whether NNKC9/41 could activate pro-FSAP in human plasma, which is a complex environment. The chromogenic substrate, S-2288, is not suitable for plasma experiments because of its low specificity and sensitivity, and therefore, we used the FSAP-specific fluorescent substrate (Ac–Ala–Lys–Nle–Arg–AMC).^35^ In normal healthy plasma, pro-FSAP is very stable and no basal FSAP activity is detectable. NNKC9/41, but not the control Ser–Ser linear peptide, induced robust activation of pro-FSAP comparable to that with histones (Figure 1D).

### Binding of NNK11/189 and NNKC9/41 to Pro-FSAP

**Figure 2:**
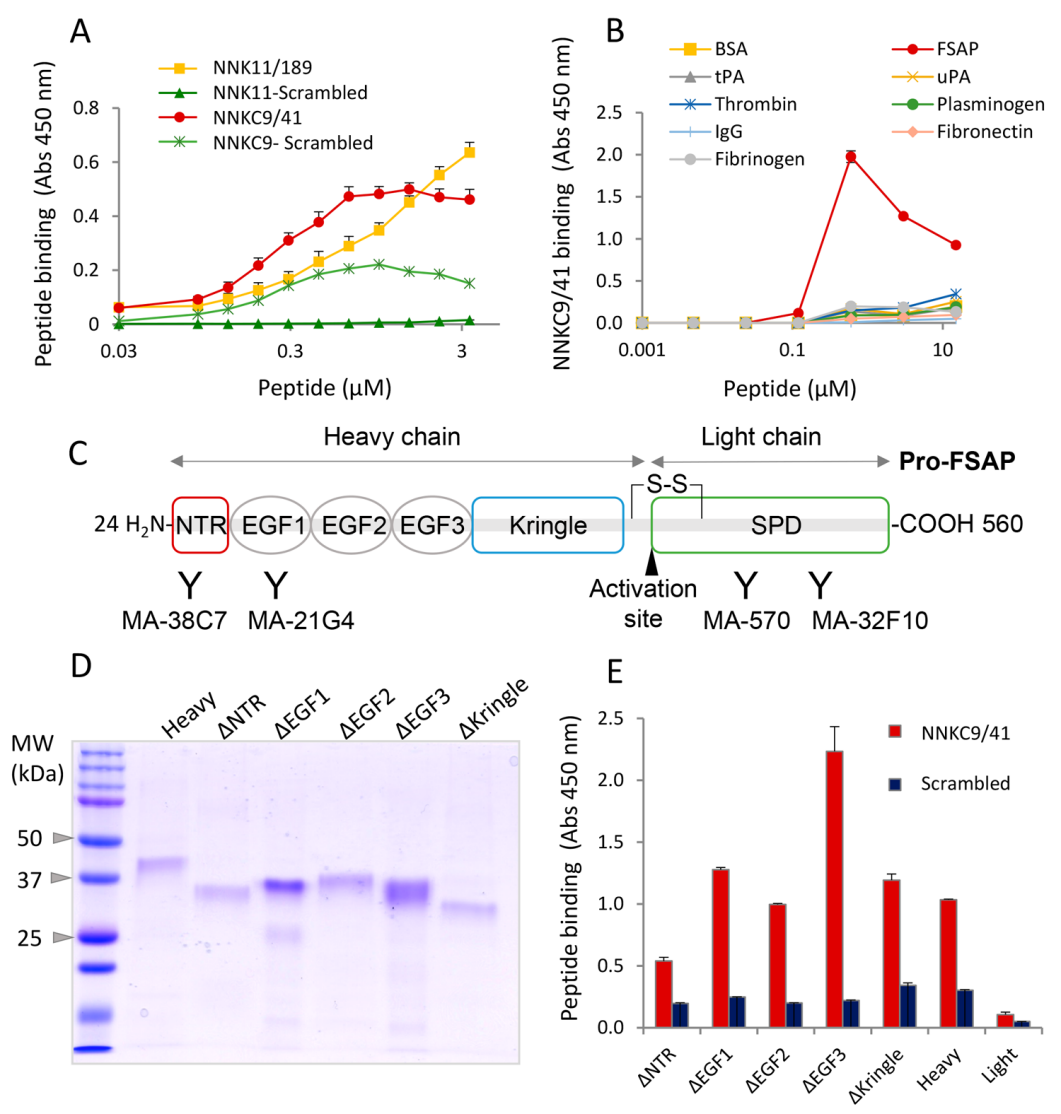
NNKC9/41 interacts with the NTR of pro-FSAP: (A) To determine peptide binding to pro-FSAP, wells were coated with 2 μg/mL pro-FSAP and biotinylated NNKC9/41 and NNK11/189 and their respective scrambled controls were added in different concentration (0–5 μM). Peroxidase-labeled streptavidin was used to detect peptide binding. (B) Same procedure as in (A) except that different proteins were immobilized and biotinylated NNKC9/41 (0–30 μM) was added and its binding was measured. (C) Schematic structure of pro-FSAP with the heavy chain consisting of an NTR, three EGF domains, and a kringle domain, followed by the light chain harboring the serine protease domain (SPD). The binding domains of antibodies used in this study are indicated. (D) Recombinant complete heavy chain (heavy) and truncated heavy chain variants ΔNTR, ΔEGF1, ΔEGF2, ΔEGF3, and ΔKringle were expressed in bacteria and analyzed by sodium dodecyl sulfate poly(acrylamide) gel electrophoresis (SDS-PAGE) followed by Coomassie staining. (E) Wells were coated with 2 μg/mL rabbit polyclonal anti-FSAP antibody and recombinant proteins (1 μg/mL) were captured. Biotinylated NNKC9/41 or the scrambled control peptide was added at 1 μM and peptide binding was measured with peroxidase-linked streptavidin. In panels (A), (B), and (E), results are shown as mean + standard error of the mean (SEM) (triplicate wells).

Next, we examined the binding properties of the peptides to pro-FSAP and other plasma proteins. N-terminally biotinylated NK11/189 and NNKC9/41 bound to immobilized pro-FSAP in a concentration-dependent manner, whereas their scrambled counterparts exhibited low binding (Figure 2A). NNKC9/41 did not bind to a selection of plasma proteins at lower peptide concentrations, although some binding was noticeable at concentrations >10 μM (Figure 2B). At these high concentrations, there was a decrease in peptide binding to pro-FSAP for unknown reasons (Figure 2B). Future studies need to be conducted with the biotinylated cyclic dimer to consolidate these results.

**Figure 3:**
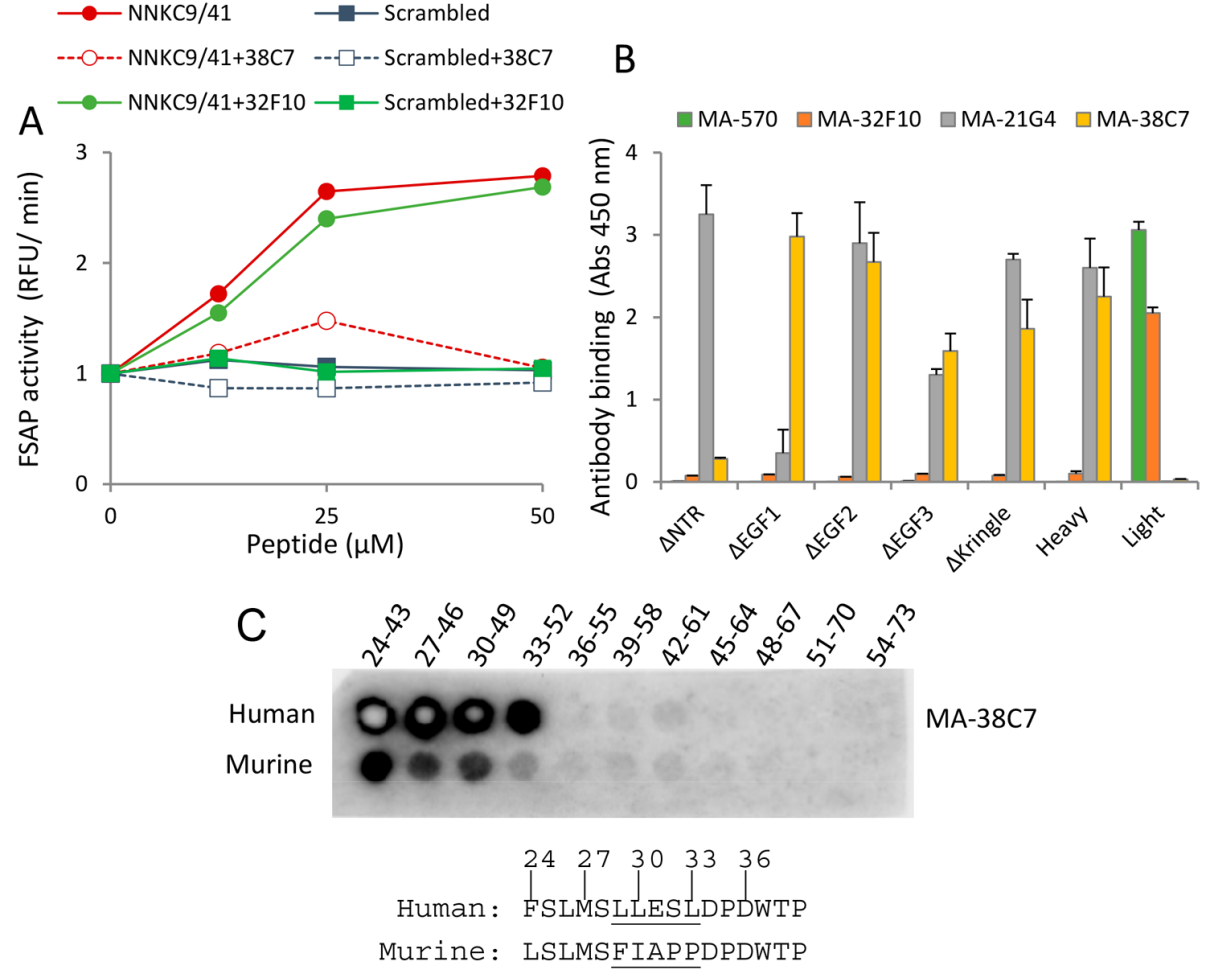
Inhibition of pro-FSAP activation by MA-FSAP-38C7 and identification of its binding epitope: (A) antibodies (6 μg/mL) were added to pro-FSAP (1.0 μg/mL) followed by NNKC9/41 or its scrambled control (0–50 μM each) and activation was determined with fluorogenic substrate Ac–Ala–Lys–Nle–Arg–AMC (80 μM) in duplicate (mean + range). (B) Wells were coated with rabbit polyclonal anti-FSAP antibody (5 μg/mL) and recombinant proteins (2 μg/mL) were captured. The indicated monoclonal antibodies were added at a concentration of 1 μg/mL, and their binding was measured with anti-mouse peroxidase-labeled secondary antibody in triplicate (mean + SEM). (C) 20-mer peptides with a three amino acid shift from the NTR (24–73) of human and mouse pro-FSAP were synthesized on nitrocellulose membranes (University of Oslo, Peptide Synthesis Facility). Membranes were blocked with 5% (v/v) bovine serum albumin (BSA), and the binding of MA-FSAP-38C7 was detected with appropriate peroxidase-coupled reagents. The MA-FSAP-38C7 binding region in pro-FSAP is amino acids 30–35, and the difference between human and mouse sequences is underlined.

To identify the domain of FSAP responsible for binding to NNKC9/41, we expressed various domain deletion mutants of pro-FSAP as His-tagged proteins in *E. coli* as described before.^36^ The N-terminus of pro- FSAP (heavy chain) consists of the intrinsically disordered N-terminal region (NTR), three EGF domains, and a kringle domain, whereas the C-terminal-light chain contains the serine protease domain (SPD) (Figure 2C). Histones have been shown to interact with the NTR of FSAP,^17^ and heparin binds to the EGF3 domain.^37^ The recombinant proteins (Figure 2D) were captured on polyclonal FSAP antibody-coated wells and were used for binding studies. NNKC9/41 exhibited high binding to the heavy chain and very low binding to the light chain/serine protease domain (SPD) and the ΔNTR mutant (Figure 2E). The scrambled peptide showed very low binding to all mutants (Figure 2E). The higher binding of the peptide to the ΔEGF3 mutant could be because this domain, when present, exerts an inhibitory influence on binding. Thus, the binding site for the NNKC9/41 was in the NTR of pro-FSAP. We then used another complementary approach to narrow down the site of NNKC9/41 binding to pro-FSAP. For this, we took advantage of a previously generated panel of monoclonal antibodies^38^ and then expanded the screen to identify antibodies that putatively modulated the effect of NNKC9/41 on pro-FSAP activation. Indeed, we identified an antibody, MA-FSAP-38C7, that completely blocked the activation of pro-FSAP by NNKC9/41 (Figure 3A), whereas a control antibody, MA-32F10, had no effect.

These experiments were performed with the FSAP-specific fluorescent substrate (Ac–Ala–Lys–Nle–Arg–AMC)^35^ as opposed to S-2238 (Figure 1C). MA-FSAP-38C7 did not influence the activity of FSAP if it was already in an activated state (data not shown). Thus, MA-FSAP-38C7, which is directed to the NTR, completely blocked pro-FSAP activation by the peptide (Figures 2C and 3B).

We then used overlapping peptide arrays from the NTR of pro-FSAP to identify the binding site for MA-FSAP-38C7. MA-FSAP-38C7 bound specifically to the LESLDP (amino acids 30–35 in full-length FSAP) sequence in the NTR region. (Figure 3C). Thus, the LESLDP sequence appears to be important for the activation of pro-FSAP. The murine sequence, which is divergent at this location, exhibited less antibody binding. Biotinylated NNKC9/41 exhibited no specific binding to this peptide array, indicating a more elaborate binding interface requiring complex structural elements of the folded protein.

### Structural Properties of NTR of FSAP

We next examined the sequence and structure of human FSAP and NTR in more detail. The predicted structure of FSAP in the Alphafold database^39^ showed that the NTR, 24–72 amino acids, is an intrinsically disordered domain followed by the well-structured EGF domains, kringle domain, and the serine protease domain (Supporting Figure S4). Other predictions of tools^40^ confirmed the intrinsically disordered nature of NTR. Based on the report about the evolution of the FSAP-encoding gene^41^ (*HABP2*) among chordates, we then compared the conservation of amino acid sequence amongst 82 different vertebrate species (Supporting Table 2). This data was mapped along with the distribution of positively and negatively charged residues over the whole FSAP molecule (Supporting Figure S4). 12/49 residues in the human NTR are negatively charged, but these are not well conserved across all species examined. Thus, human NTR is a likely interaction site for histones and NNKC9/41, but the activation mechanism might be different in other species. Intrinsically disordered domains can interact with multiple partners because of their structural plasticity and are often involved in regulatory processes in a structure-independent manner.^42^

### Pro-FSAP Activation by NNKC9/41 in Human Plasma

Next, we characterized the effects of the peptide on pro-FSAP activation in plasma in detail. NNKC9/41 activated pro-FSAP in plasma anticoagulated with hirudin at plasma dilutions ranging from 1:2 to 1:12. However, in plasma anticoagulated with citrate, the effect of NNKC9/41 was completely abolished at lower dilution (1:2) and weak at higher dilution (1:12) (Supporting Figure S5). The inhibitory effect of citrate was reversed by recalcification of plasma (1:12) (data not shown), which shows that Ca^2+^ is required for the optimal pro-FSAP activation by the peptide. In general, both types of plasma exhibited lower pro-FSAP activation in 1:2 diluted plasma because the concentration of endogenous plasma inhibitors is higher at this dilution. Pro-FSAP activation had a longer lag time with the peptide (>15 min) compared to histones (Figure 1D). Western blotting of hirudin plasma and citrate plasma activated with NNKC9/41 showed the emergence of an FSAP-inhibitor complex band that is a proxy marker for the activation of pro-FSAP in plasma (Supporting Figure S6).

**Figure 4:**
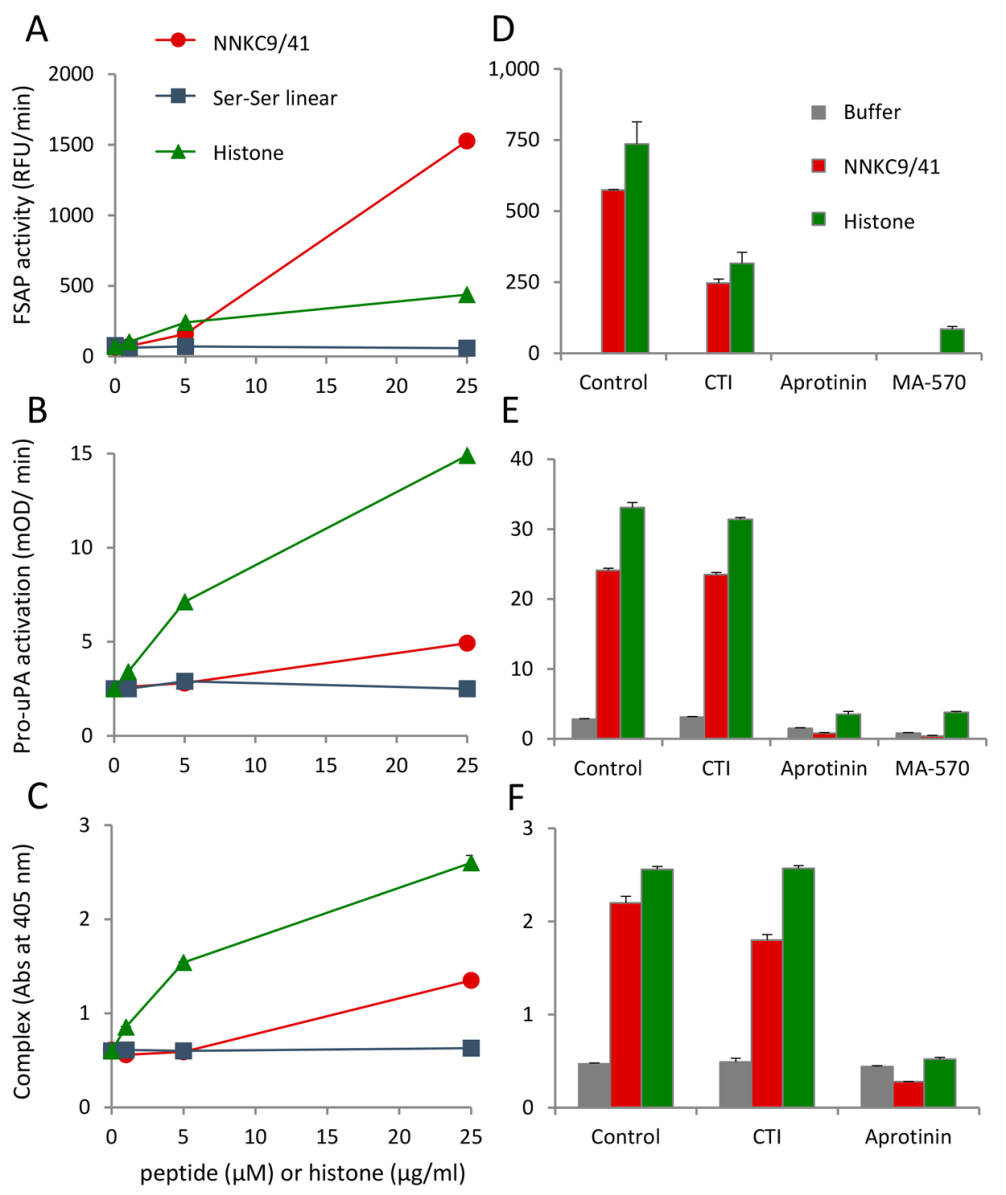
Comparison of different methods to measure pro-FSAP activation in plasma: (A) hirudin plasma (1:12 dilution) was incubated with histones (0–25 μg/mL), NNKC9/41, or its Ser–Ser linear control (0–25 μM) and the fluorogenic substrate turnover (Ac–Ala–Lys–Nle–Arg–AMC) was monitored. (B) From the same experiment as in (A) after 1 h incubation at 37 °C, samples were captured on anti-FSAP coated wells and the conversion of added pro-uPA to active uPA was measured by determining the turnover of the chromogenic substrate PNAPEP1344 (25 μM). (C) From the same experiment as in (A) after 1 h incubation at 37 °C, samples were captured on anti-FSAP coated wells and α2-antiplasmin antibody was used to detect the formation of FSAP−α2-antiplasmin complexes. (D–F) Hirudin plasma (1:12 dilution) was activated with histones (20 μg/mL) or NNKC9/41 (25 μM) in the presence of CTI (80 μg/mL), aprotinin (100 μg/mL), or MA-570 (20 μg/mL) followed by analysis as described in (A), (B), and (C), respectively. In (F), the samples with MA-FSAP-570 were not analyzed since this antibody would interfere in the sandwich ELISA. For panels (A)–(F), results are shown as mean + range of duplicates wells and the results were replicated in plasma samples from three donors.

To consolidate the results and exclude any artefacts, we used two additional methods to demonstrate that NNKC9/41 activated pro-FSAP in plasma; (i) immunocapture of pro-FSAP from plasma, its activation by the peptide, and the conversion of the substrate pro-uPA to uPA and (ii) the formation of FSAP–alpha2–antiplasmin complexes in plasma as a readout for pro-FSAP activation and its subsequent inhibition. A concentration-dependent effect of NNKC9/41 and histones demonstrated that the peptide was active in both these assays (Figure 4A–C). The differences in the dynamics of the activation process and the properties of the different assays account for the fact that the peptide has a stronger effect than histones on fluorogenic substrate turnover (Figure 4A) but is weaker than histones in the other two assays (Figure 4B,C). For example, pro-FSAP activation can be similar in two plasma samples using the fluorescent substrate but complex formation with alpha2–antiplasmin is influenced by the concentration of C1 inhibitor present as established before.^43^ The use of different methods to demonstrate pro-FSAP activation in plasma underscores the robustness and reproducibility of the effects of the peptide.

The contact pathway of blood coagulation is activated by negatively charged surfaces,^44^ and we also tested whether it was involved in any way in the pro-FSAP activation process. Blocking the contact pathway with the FXIIa inhibitor, corn trypsin inhibitor (CTI), did not influence the activation of pro-FSAP by the peptide (Figure 4D–F). The small inhibitory effect of CTI (80 μg/mL) is because of its direct inhibitory effect on active FSAP (data not shown). The kallikrein inhibitor PKS1-527 had no effect,^43^ but aprotinin, a general serine protease inhibitor, blocked pro-FSAP activation/activity by directly blocking FSAP (Figure 4D–F). MA-FSAP-570, a known inhibitory antibody against FSAP, also blocked the activity/activation of FSAP/pro-FSAP (Figure 4D,E). Hence, we can exclude the involvement of the contact pathway in the pro-FSAP activation by the peptide.

The next question was whether the peptide was activating a mechanism common to many zymogens^31^ or whether it was selective for pro-FSAP. NNKC9/41 did not influence the activity of plasminogen, pro-urokinase, factor XII, prothrombin, factor X, and their respective active enzymes (Supporting Figure S7). This is also in line with the observation that it did not bind to any other plasma proteins tested, except FSAP (Figure 2B). Thus, the peptide showed very high selectivity for pro-FSAP activation. NNKC9/41 did not alter the activity of the recombinant serine protease domain (SPD) of FSAP (Supporting Figure S7A) indicating that it has no effect on, already, active FSAP.

**Figure 5:**
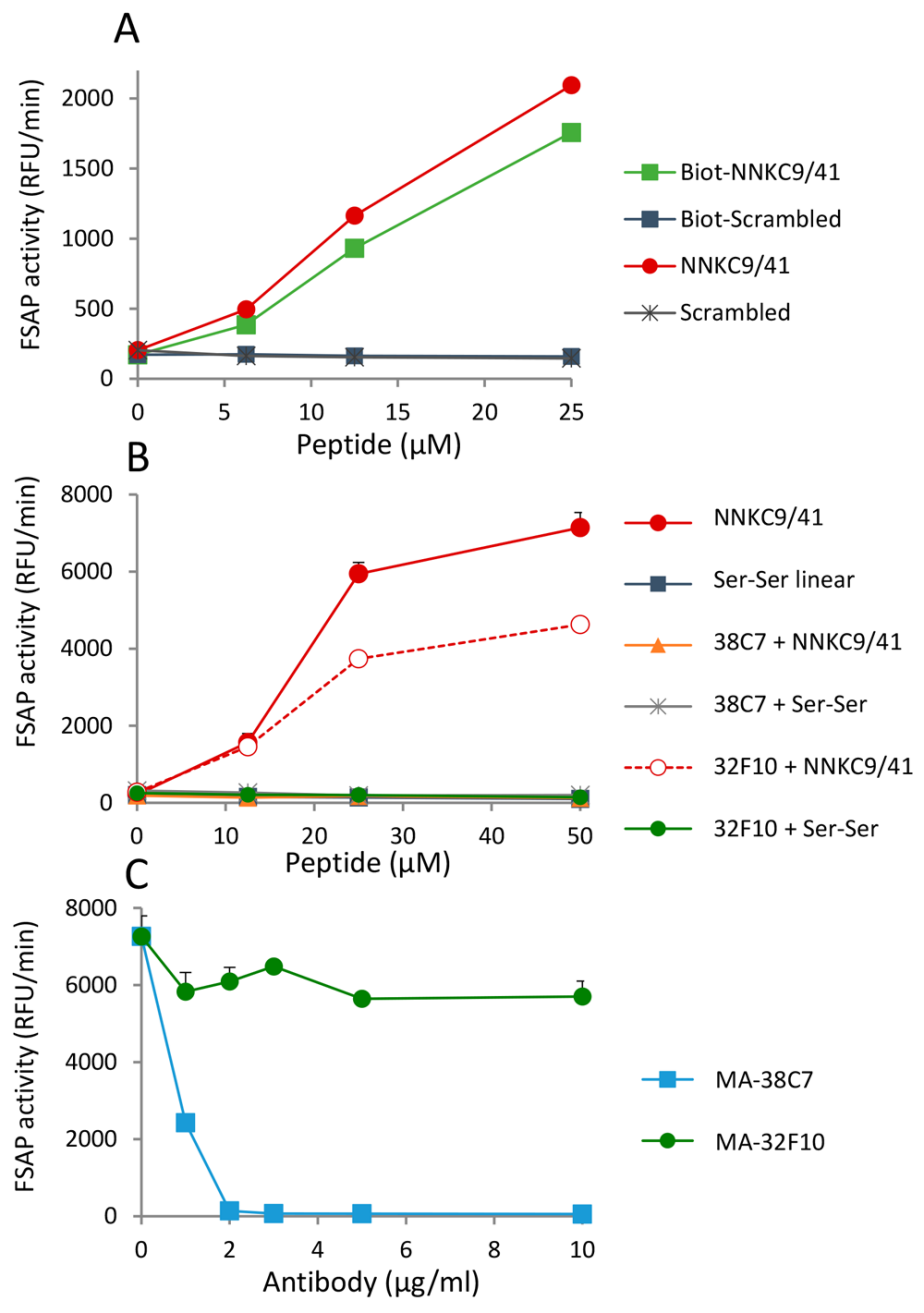
Characteristics of pro-FSAP activation in plasma by NNKC9/41: (A) neutravidin-coated wells were used to capture biotinylated NNKC9/41 or scrambled control peptide (0–25 μM). Hirudin plasma (1:10) was incubated in the wells for 1 h at 37 °C to allow for binding and activation of pro-FSAP. In parallel, wells were coated with rabbit polyclonal anti-FSAP antibody (2 μg/mL) and plasma was added to immobilize FSAP in the presence of solution phase NNKC9/41 or its scrambled control (0–25 μM). Fluorogenic substrate turnover (Ac–Ala–Lys–Nle–Arg–AMC) was monitored. (B) Plasma was incubated with NNKC9/41 and Ser–Ser control (0–50 μM each) in the absence of any antibody or in the presence of MA-FSAP-38C7 or MA-FSAP-32F10 (5 μg/mL) and the FSAP activity was monitored. (C) Plasma was incubated with NNKC9/41 (25 μM) in the presence of MA-FSAP-38C7 or MA-FSAP-32F10 (0–10 μg/mL) and the FSAP activity was monitored. For (A)–(C), results are shown as mean + range of duplicate wells.

In the hemostasis cascade, zymogen activation often requires the association of the various components on a surface and pro-FSAP has been shown to be activated by positively charged surfaces.^18^ To mimic this surface-dependent process, we immobilized biotinylated NNKC9/41 on neutravidin-coated plates. After capture and preincubation with hirudin plasma, robust activation of pro-FSAP was observed, but this was not the case with the biotinylated scrambled peptide (Figure 5A). In a corollary of the above experiment, pro-FSAP was captured from plasma on anti-FSAP antibody-coated wells and NNKC9/41 and its scrambled counterpart were added to the wells to activate the immobilized pro-FSAP. Only NNKC9/41 was found to activate pro-FSAP (Figure 5A), confirming the activity of this peptide in different assay configurations also with N-terminal biotinylation.

Antibody MA-FSAP-38C7 was then tested for its ability to modulate pro-FSAP activation in plasma. Dose–response analysis showed that the MA-FSAP-38C7 antibody was a potent inhibitor of the peptide-mediated activation of pro-FSAP in plasma (Figure 5B,C), whereas MA-FSAP-32F10 showed a much weaker effect. These antibodies and their Fab fragments showed the same effect when histones were used as activators of pro-FSAP instead of the peptide (Supporting Figure S8).

**Figure 6:**
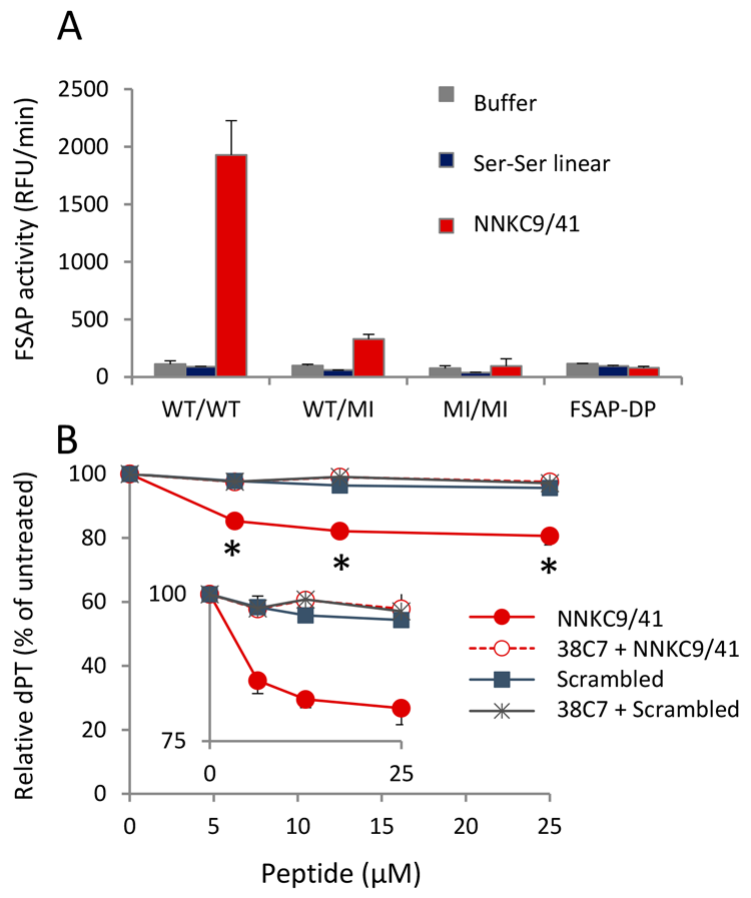
Pro-FSAP activation and clotting in plasma by NNKC9/41: (A) citrate plasma (1:12 dilution) from donors with WT/WT, WT/MI, and MI/MI genotypes and WT-FSAP-deficient plasma (FSAP-DP) were compared. Substrate turnover was monitored after addition of NNKC9/41 or the Ser–Ser linear peptide (25 μM each). Results are shown as mean + range of duplicate wells. (B) Pooled prothrombin-deficient citrated plasma (1:5 dilution) was recalcified with 10 mM CaCl_2_ and incubated with NNKC9/41 or scrambled control peptide (0–25 μM each) for 60 min at 37 °C in the presence or absence of the FSAP-inhibitory MA-FSAP-38C7 (5 μg/mL). Initiation of clotting was achieved by adding the tissue factor/phospholipid and prothrombin complex concentrate. Clotting time was measured as diluted prothrombin time (dPT) in seconds. Data are the mean of three independent experiments. The relative dPT in % of the untreated control is presented as mean ± standard error (SE) (*p <0.05, two-way analysis of variance (ANOVA) and Bonferroni post-test). The inset shows a magnified *y*-axis.

To consolidate the above results, we also performed studies on FSAP-depleted plasma and on plasma from persons who were heterozygous or homozygous for the MI-SNP (all anticoagulated with citrate and diluted 1:12). In FSAP-depleted plasma, no turnover of the substrate was observed in the presence of the NNKC9/41. Similarly, the peptide mediated high substrate turnover in WT/WT plasma, low turnover in WT/MI plasma, and none in MI/MI plasma (Figure 6A). These results further underscore the direct, selective, and specific effect of the peptide on the activation of endogenous pro-FSAP in plasma.

### Effect of NNKC9/41 on Hemostasis

We then addressed the question of whether the activation of pro-FSAP in plasma with NNKC9/41 leads to any hemostasis-related effects. We have previously shown that FSAP promotes coagulation by inactivating tissue factor pathway inhibitor (TFPI),^38^ which is an inhibitor of the tissue factor-dependent pathway of blood coagulation. As described above, the use of citrate plasma for clotting experiments was problematic because the peptide did not function optimally in undiluted citrate plasma and, after calcification, the peptide had a long lag time for pro-FSAP activation, whereas clotting is a very fast process. To overcome these issues, we performed a clotting assay where the peptide was added to prothrombin-deficient plasma in the presence of Ca^2+^ to allow for pro-FSAP activation but without any premature clotting of the plasma. A prothrombin-containing concentrate was then added to enable tissue factor/phospholipid-triggered clotting. NNKC9/41 had a significant effect on lowering the clotting time, and this effect was reversed in the presence of MA-FSAP-38C7 (Figure 6B). Thus, NNKC9/41 activated pro-FSAP and MA-38C7 inhibited it, leading to functional consequences for clotting. It should be noted that the antibody also inhibits the effect of FSAP on TFPI as shown previously,^38^ indicating that the NTR of FSAP also binds to TFPI.

### Conclusions

In the phage library, the peptide was likely to be displayed as a cyclic peptide within the pIX protein. Since five copies of the pIX protein are present on each phage, some intermolecular disulfide bond formation is feasible. It is also possible that during the binding-based phage selection process, the linear or monocyclic peptide on phages had sufficient binding affinity to enable selection. The antiparallel cyclic dimer is only assembled in the synthetic linear peptide preparation but is probably not found on phages.

The contact pathway of blood coagulation, which involves the activation of FXII and PK, is stimulated by negatively charged molecules.^44^ Pro-FSAP activation follows a divergent pattern in that it is activated by positively charged macromolecules. In a model of pro-FSAP activation proposed by Yamamichi et al.,^19^ disruption of charge-based interaction between NTR and EGF3 domains of pro-FSAP by histones leads to intermolecular dimerization between two pro-FSAP molecules followed by autoactivation. The antiparallel cyclic-dimeric peptide, which theoretically has two binding sites in opposite orientations, may be ideally suited for promoting FSAP dimerization and activation. This together with the concentration of negatively charged residues in the intrinsically disordered NTR of human FSAP points to the existence of a unique zymogen activation mechanism among blood proteases that needs further characterization.

This peptidic activator of pro-FSAP, as well as its interaction sequence in pro-FSAP, will be a useful tool to establish the molecular details of FSAP zymogen activation. This information can be used to develop inhibitors of FSAP activation that will help to elucidate the biological role of FSAP and develop novel therapeutic strategies.

## Materials and Methods

### Peptides

Peptides were synthesized by GenScript (Piscataway, New Jersey) or JPT (Berlin, Germany). For a selection of peptides, the sequence and the number of batches tested in this work are indicated in parentheses in Supporting Table 1. Where specified, the peptides were biotinylated at the N-terminus by the vendor. All peptides were dissolved in dimethyl sulfoxide (DMSO) to provide maximal solubility.

### Helper Phages, Bacterial Strains, and Construction of Peptide Phage Libraries

M13K07 was purchased from GE Healthcare (Uppsala, Sweden), whereas DeltaPhage was prepared as described.^45^*E. coli* strains SS320 and XL1-Blue were purchased from Lucigen Corp. (Middleton, Wisconsin) and Life Technologies (Carlsbad, California), respectively. A library template phagemid was prepared by inserting a short oligo containing two consecutive stop codons (ocher/opal) into the *Nco*I/*Bam*HI expression cassette of the pGALD9ΔL phagemid^46^ using standard methods. Using this template, a linear random 11-mer (NNK11) and a Cys-constrained random 9-mer (NNKC9) peptide library fused to pIX was constructed based on Kunkel mutagenesis essentially as described.^47^ The final *E. coli* SS320 transformation frequency-based library sizes were about 1 × 10^10^ unique clones, and the libraries were prepared with high-valence peptide display by rescue with DeltaPhage.

### Phage Selection with Human FSAP

Tubes and streptavidin-coated Dynabeads (Thermo Fischer Scientific, Oslo, Norway) were blocked with PBS with 4% (w/v) skim milk powder. Approximately 1 × 10^13^ virions of library and 1–10 μg (R1, 10 μg; R2, 3:1 μg) of biotinylated pro-FSAP were incubated with the appropriate amount of Dynabeads for 60 min at room temperature (RT). The beads/protein/phage complexes were separated from the supernatant using a magnetic rack and washed with PBS-T20 (0.1% (v/v) Tween-20 in phosphate-buffered saline (PBS)). The complexes were then incubated with an excess amount of unbiotinylated pro-FSAP for 60 min at RT to remove phages bound with low-affinity and retain only high-affinity binding clones. The beads/protein/phage complexes were then separated from the supernatant containing the soluble FSAP using a magnet rack and washed with PBS-T20 (0.1% (v/v) Tween-20 in PBS) and with PBS pH 7.4. This was applied to both libraries and in all rounds. The remaining phages were then eluted from the beads with 500 μL of 100 mM triethylamine (pH 11) and neutralized in Tris–HCl pH 7.4.

### Bacteria Preparation, Library Amplification, and Phage Particle Preparation

*E. coli* XL1-Blue bacteria were grown in 2xYT-TAG (30 μg/mL tetracycline, 100 μg/mL ampicillin, and 0.1 M glucose) medium at 37 °C overnight and used to make a fresh rescue culture. Phages eluted from R1 were added to log-phase cells in 2xYT-TAG, followed by incubation at 37 °C. After centrifugation, the pellet was resuspended, and the solution was plated onto 2xYT-TAG Q-trays and incubated at 30 °C overnight. The next day, cells were scraped from the agar plates with a total volume of 20 mL of 2xYT. The scraped material was used to reinoculate 50 mL of 2xYT-TAG medium at 37 °C until it reached an OD600 of 0.2 before superinfection (multiplicity of infection 20) with the M13K07 helper phage. Cells were pelleted by centrifugation, and the pellet was gently resuspended in 50 mL of prewarmed 2xYT AK (100 μg/mL ampicillin and 50 μg/mL kanamycin) and incubated to produce phage particles displaying peptides at the low valence. The culture was centrifuged at 10,000 rpm for 10 min, and the supernatant was sterile-filtrated using a 0.2 μm filter. The phage particles were purified and concentrated by PEG/NaCl precipitation as described.^48^ Virion concentration was determined by the following formula: virions/mL = [(*A*_269nm_ – *A*_320nm_) × 6.083 × 10^16^]/genome size.^49^

### Binding Studies with Biotinylated Peptides

Wells were coated with anti-FSAP polyclonal antibodies (5 μg/mL). Plates were washed with TBS (25 mM Tris–HCl (pH 7.5) and 150 mM NaCl) containing 0.2% (w/v) Tween-20 (TBS-T) and were blocked with TBS containing 3% (w/v) BSA (Sigma). Recombinant FSAP proteins were captured, and biotinylated peptides were added to allow binding. Subsequently, the plates were washed, and bound peptides were detected with peroxidase-coupled streptavidin. Nonspecific binding to BSA was subtracted from binding to the test protein to calculate specific binding.

### Recombinant Protein Expression

FSAP cDNA was derived from human liver RNA and subsequently cloned into the pASK-IBA33plus vector (IBA-Lifesciences, Goettingen, Germany). This vector contains a C-terminal 6xHIS tag. The construct of the recombinant N-terminal FSAP comprising amino acids 24-313 was used as template to generate mutants with the following amino acids deletions: ΔNTR (24–72), ΔEGF1 (73–109), ΔEGF2 (111–148), ΔEGF3 (150–188), and ΔKringle (193–276). The serine protease domain construct comprises amino acids 292–560, and its expression has been described before.^50^

Proteins were expressed in BL21-Gold (DE3) in inclusion bodies. Washed inclusion bodies were resuspended in 100 mM Tris, 150 mM NaCl, 5 mM 2-mercaptoethanol, and 8 M urea pH 8 for 1 h followed by centrifugation at 14,000 rpm for 10 min. After purification on a Ni-NTA-agarose column, the protein was diluted to a concentration of 0.1 mg/mL and dialyzed in 100 mM Tris, 150 mM NaCl, 5 mM 2-ME, and 8 M urea pH 8 for 1 h at room temperature. This was followed by dialysis in 20 mM Tris, 10% (v/v) glycerol, and 4 M urea, pH 8 at 4 °C overnight and finally in 20 mM Tris pH 8 and 10% (v/v) glycerol overnight at 4 °C. The protein was cleared for any precipitation method, and the concentration was determined by Bio-Rad reagents using BSA as a standard. Protein quality was checked by Coomassie blue staining of SDS-PAGE gels.

### Activation of Endogenous Pro-FSAP in Plasma

Human citrate plasma (0.38% w/v) or hirudin plasma (25 μg/mL Lepirudin) was obtained from five healthy donors. Plasma was diluted at 1:12 and mixed with various test substances that potentially activate or inhibit pro-FSAP. Ac–Ala–Lys–Nle–Arg–AMC (amino–methyl–coumarin) was used as a sensitive and specific substrate for FSAP.^35^ Hydrolysis of the fluorogenic substrates was measured using a Synergy HI plate reader with excitation at 320 nm and emission at 460 nm at 37 °C for 60 min. The maximal velocity was calculated from the linear part of the progress curve. In limited experiments, the second-generation fluorogenic FSAP substrate (Ac–Pro–dTyr–Lys–Arg–AMC)^34^ was used. Pro-uPA activation, formation of FSAP−α2-antiplasmin, and western blotting with anti-FSAP antibodies were also used to monitor pro-FSAP activation.

### Pro-FSAP Enzyme Activity Assay

Pro-FSAP was isolated from human plasma as described before.^14^ The content of the zymogen form varied from 50% to 90% due to autoactivation during the purification process. Histones, isolated from the calf thymus, were obtained from Sigma-Aldrich (Oslo, Norway). FSAP activity assays were performed as described previously.^51^ In brief, microtiter wells were blocked with TBS (25 mM Tris–HCl, pH 7.5, and 150 mM NaCl) containing 3% (w/v) BSA for 1 h and washed with TBS-T. The standard assay consisted of TBS-T with 0.3% (w/v) BSA and CaCl_2_ (2 mM) 1 μg/mL (15 nM) plasma purified pro-FSAP and 250 μM chromogenic substrate S-2288 (d-Ile-l-Pro-l-Arg-*p*-nitroaniline dihydrochloride) (Haemochrome Diagnostica, Essen, Germany) and was followed at 37 °C at 405 nm in a microplate reader Synergy HI plate reader (BioTek Instruments, Winooski). In some experiments, a fluorescent substrate of FSAP^35^ was used.

### Effect of FSAP-Activating Peptide NNKC9/41 on the Extrinsic Pathway of Coagulation

Prothrombin-deficient citrated pool plasma (Haemochrom Diagnostica, diluted 1:5 in HBS, pH 7.4) was recalcified with 10 mM CaCl_2_ and incubated with peptides for 60 min at 37 °C in the presence or absence of the FSAP-inhibitory MA-FSAP-38C7 (5 μg/mL). Initiation of clotting was achieved by adding 100 μL of tissue factor/phospholipids, Thromborel (Siemens Healthcare, Marburg, Germany), 1:3000 prediluted in HBS and, prior to use, adjusted to 0.3 IU/mL FII by addition of a prothrombin complex concentrate (Ph. Eur. quality). The final concentrations in the clotting assay were 1:8 diluted plasma, 1:7500 Thromborel, and 0.125 IU/mL prothrombin. Clot formation was measured optically at 405 nm in a plate reader (Tecan, Crailsheim, Germany). The time from starting the reaction to 50% maximum clot turbidity was measured as diluted prothrombin time (dPT) in seconds.

### Isolation of the Cyclic Dimer from the Linear Peptide Preparation

NNKC9/41 peptide was obtained by Fmoc solid-phase peptide synthesis in a Liberty blue microwave peptide synthesizer (CEM Corporation, Matthews, NC) as previously described.^52^

The cyclic dimer was formed by incubation of the peptide in 50 mM Tris + 150 mM NaCl (pH 8.45) for 24 h at RT. Then, the mixture was subjected to fractionation in a reversed-phase Aeris XB-C18 column (Phenomenex) of dimensions 10 mm × 250 mm, a particle size of 5 μm, and a pore size of 100 Å. The following gradient of acetonitrile is used for elution: 0/5, 5/15, 45/30, 55/80 (retention time, min/%B); solvent A, 0.1% TFA in water; and solvent B, 0.1% TFA in acetonitrile. The fractions were tested in a pro-FSAP activation assay using hirudin plasma and the fluorescent substrate as described above and compared to the UV absorption values at 280 nm.

### Mass Spectrometry of the Cyclic Dimer

The cyclic dimer purified by HPLC was analyzed without any modification and also after digestion with LysC. A 15 μL aliquot was used for mass spectrometry analysis, as follows: the sample was measured using an Orbitrap Elite Hybrid mass spectrometry system (Thermo Fisher Scientific, Bremen, Germany) online coupled to a U3000 RSLCnano (Thermo Fisher Scientific) employing an Acclaim PepMap analytical column (75 μm × 500 mm, 2 μm, 100 Å, Thermo Fisher Scientific) at a flow rate of 250 nL/min. Using a C18 μ-precolumn (0.3 mm × 5 mm, PepMap, Dionex LC Packings, Thermo Fisher Scientific), samples were preconcentrated and washed with 0.1% TFA for 5 min at a flow rate of 30 μL/min. The subsequent separation was carried out using a binary solvent gradient consisting of solvent A (0.1% FA) and solvent B (86% ACN, 0.1% FA). The column was initially equilibrated in 5% B. In the first elution step, the percentage of B was increased from 5 to 15% in 5 min, followed by an increase from 15 to 40% B in 30 min. The column was washed with 95% B for 4 min and re-equilibrated with 5% B for 19 min. The mass spectrometer was equipped with a nanoelectrospray ion source and distal-coated SilicaTips (FS360-20-10-D, New Objective, Woburn, Massachusetts). The instrument was externally calibrated using standard compounds (LTQ Velos ESI Positive Ion Calibration Solution, Pierce, Thermo Scientific, Rockford). The system was operated using the following parameters: spray voltage, 1.5 kV; capillary temperature, 250 °C; S-lens RF level, 68.9%. XCalibur 2.2 SP1.48 (Thermo Fisher Scientific) was used for data-dependent tandem mass spectrometry (MS/MS) analyses. Full scans ranging from *m*/*z* 370 to 1700 were acquired in the Orbitrap at a resolution of 30,000 (at *m*/*z* 400) with automatic gain control (AGC) enabled and set to 10^6^ ions and a maximum fill time of 500 ms. Up to 20 multiply-charged peptide ions were selected from each survey scan for collision-induced fragmentation (CID) in the linear ion trap using AGC set to 10,000 ions and a maximum fill time of 100 ms. For MS/MS fragmentation, a normalized collision energy of 35% with an activation *q* of 0.25 and an activation time of 30 ms was used. FreeStyle 1.8 SP1 (Thermo Fisher Scientific) was used for spectra visualization and deconvolution.

### Modeling of FSAP Structure and Sequence Comparison across the Phylogenic Tree

The predicted structure of human FSAP was taken from the Alphafold database.^39^ Intrinsically disordered domains in FSAP were predicted using multiple programs as described before.^40^ An orthology assignment of plasminogen activation system members in chordates indicated the presence of canonical *HABP2* gene in all subgroups except lampreys.^53^ Using the human sequence for HABP2 as a query, a homologous BLAST search among these species was performed using blastp ncbi-blast-2.12.0+.^54^ A total of 81 sequences (Supporting Table 2) were aligned and submitted to ConSurf^55^ with default parameters, and the conservational score of each amino acid was predicted by the Bayesian method.

### Statistical Analysis

All experiments relating to the activation of purified pro-FSAP or plasma were performed with different batches of synthetic peptides and different donors. Each experiment was performed in duplicates or triplicates, and the results were shown as mean ± range or mean ± SEM, respectively. In the plasma clotting assays, results from three independent experiments were pooled and shown as mean ± SEM, and the statistical analysis was performed using two-way analysis of variance (ANOVA) followed by the Bonferroni test using Graphpad prism.

## Supporting Information Available

Characterization of NNKC9/41 cyclization states; structural modeling of FSAP and homology across species; effect of anticoagulants on pro-FSAP activation in plasma; lack of the effect of NNKC9/41 on activation of other plasma protein zymogens (PDF)

## Notes

**Data sharing statement**: The data that support the findings of this study are available from the corresponding author upon reasonable request.
