# Supplementary material for: Identification of a Phage Display-Derived Peptide Interacting with the N-Terminal Region of Factor VII Activating Protease (FSAP) Enables Characterization of Zymogen Activation
Source: ACS Chem Biol. 2022 Sep 7;17(9):2631–42. doi: 10.1021/acschembio.2c00538 (PMC9486805; doi:10.1021/acschembio.2c00538)
Supplement: Supplementary file 1 — cb2c00538_si_001.pdf [file cb2c00538_si_001.pdf]

Supporting information

Identification of a phage display-derived peptide interacting with the N-terminal region of Factor VII activating protease (FSAP) enables characterization of zymogen activation.

Sebastian Berge-Seidl[1#], Nis Valentin Nielsen[1#], Armando A. Rodriguez Alfonso[2#], Michael Etscheid[3], Sai Priya Sarma Kandanur[1], Bengt Erik Haug[4], Maria Stensland[1], Bernd Thiede[5], Merve Karacan[2], Nico Preising[2], Sebastian Wiese[2], Ludger Ständker[2], Paul J. Declerck[6], Geir Åge Løset[1, 7] and Sandip M. Kanse[1*].

#Joint 1st authors

[1]Oslo University Hospital and Medical Faculty, University of Oslo, 0372 Oslo, Norway. [2]Ulm University Medical Center, 89081 Ulm, Germany, [3]Paul Ehrlich Institute, 63225 Langen, Germany. [4]Department of Chemistry and Center for Pharmacy, University of Bergen, 5007 Bergen, Norway. [5]Department of Biosciences, University of Oslo, 0371 Oslo, Norway. [6]Department of Pharmaceutical and Pharmacological Sciences, Katholieke Universiteit 3000 Leuven, Belgium. [7]Nextera, 0349 Oslo, Norway.

***Correspondence:** Sandip M. Kanse, Institute for Basic Medical Sciences, University of Oslo, 0172, Oslo, Norway. E-mail: sandip.kanse@medisin.uio.no

Supplementary Table 1: Overview of the peptides

**Sequence**	**Name**	**Source and number of syntheses**
**CRGAMWMYKRC**	NNKC9/41 (linear)	Genescript (4)<br>JPT (1)<br>In house (1)
**CRGAMWMYKRC**	NNKC9/41<br>Mono-cyclic Cys-Cys	Genescript (3)<br>JPT (1)
**cyclo(SRGAMWMYKRS)**	Ser-Ser cyclic	Genescript (1)
**SRGAMWMYKRS**	Ser-Ser linear	Genescript (2)<br>JPT (1)
**RCGARCYMKWM**	NNKC9/41<br>Scrambled	Genescript (2)
**IDCLMQNAGSA**	NNK11/189	Genescript (1)
**NALAQCDGSIM**	NNK11/189<br>Scrambled	Genescript (1)
**CRGAMWMYKRC**<br>**CRGAMWMYKRC**	Anti-parallel<br>Cyclic dimer	Purified by HPLC from NNKC9/41

Supplementary Table 2: HABP2 sequence from the following species were aligned.

Species	Chordata
Carcharodon carcharias	Cartilaginous species
Chiloscyllium plagiosum	
Chiloscyllium punctatum	
Callorhinchus milii	
Amblyraja radiata	
Scyliorhinus canicula	
Scyliorhinus torazame	
Rhincodon typus	
Polypterus senegalus	Bichirs
Erpetoichthys calabaricus	
Notolabrus celidotus	Teleosts
Seriola dumerili	
Toxotes jaculatrix	
Chelmon rostratus	
Cheilinus undulatus	
Plectropomus leopardus	
Colossoma macropomum	
Scatophagus argus	
Electrophorus electricus	
Megalops cyprinoides	
Latimeria chalumnae	Coelacanth
Protopterus annectens	Lungfish
Xenopus tropicalis	Amphibians
Bufo gargarizans	
Rana temporaria	
Xenopus laevis	
Nanorana parkeri	
Bufo bufo	
Hymenochirus boettgeri	
Polyodon spathula	Ray-finned fishes
Acipenser ruthenus	
Amia calva	
Homo sapiens	Mammals
Pan paniscus	
Gorilla gorilla gorilla	
Hylobates moloch	
Nomascus leucogenys	
Piliocolobus tephrosceles	
Rhinopithecus bieti	
Rhinopithecus roxellana	
Macaca mulatta	
Macaca fascicularis	

Species	Chordata
Anolis carolinensis	Lizards
Podarcis muralis	
Lacerta agilis	
Gekko japonicus	
Zootoca vivipara	
Sceloporus undulatus	
Pogona vitticeps	
Varanus komodoensis	
Python bivittatus	Snakes
Crotalus tigris	
Protobothrops mucrosquamatus	
Pantherophis guttatus	
Thamnophis sirtalis	
Thamnophis elegans	
Notechis scutatus	
Pseudonaja textilis	
Terrapene carolina triunguis	Turtles
Chrysemys picta bellii	
Trachemys scripta elegans	
Mauremys reevesii	
Chelydra serpentina	
Gopherus evgoodei	
Chelonia mydas	
Dermochelys coriacea	
Mauremys mutica	
Chelonoidis abingdonii	
Alligator mississippiensis	Crocodilians
Crocodylus porosus	
Alligator sinensis	
Gavialis gangeticus	
Falco peregrinus	Birds
Falco naumanni	
Falco cherrug	
Charadrius vociferus	
Aramus guarauna	
Ciccaba nigrolineata	
Fulmarus glacialis	
Alca torda	
Cuculus canorus	
Rynchops niger	

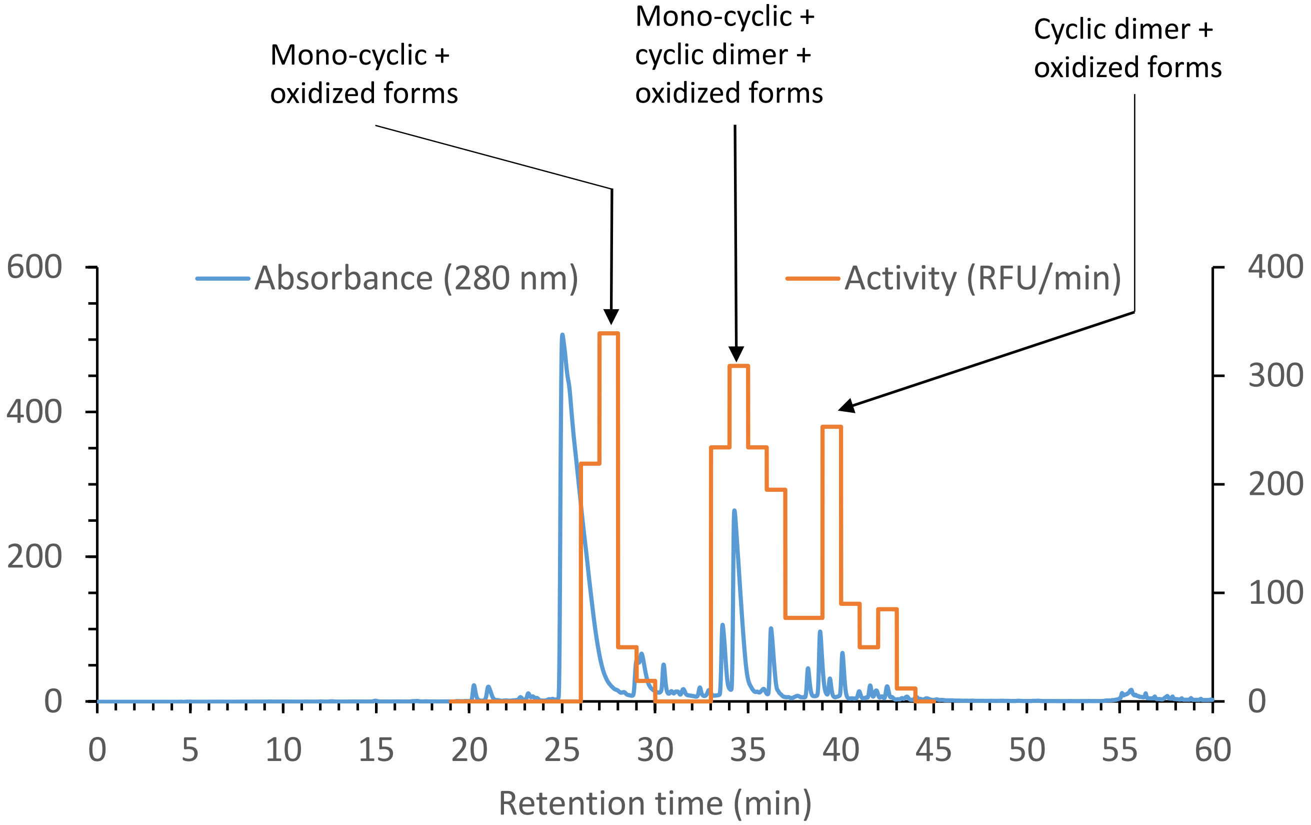

Fig S1. Analysis of repurified synthetic NNKC9/41: NNKC9/41 was dissolved in DMSO and purified by HPLC. Fractions 19-45 were tested for activation of pro- FSAP in hirudin plasma. Peak active fractions were analyzed by LC-MS. The monoisotopic masses (M) corresponded to the molecular masses of NNKC9/41 of the mono-cyclic (m/z= 468.207 (3+) and 701.806 (2+); M= 1401.598 Da), oxidized mono-cyclic (m/z= 473.539 (3+) and 709.804 (2+); M= 1417.594 Da), cyclic dimer (m/z= 561.647 (5+), 701.807 (4+), 935.408 (3+), and 1402.603 (2+) ; M = 2803.224 Da), oxidized cyclic dimer (m/z= 564.846 (5+), 705.806 (4+), 940.739 (3+); M=2819.192 Da), and di-oxidized cyclic dimer (m/z=568.045 (5+), 709.805 (4+), 946.070 (3+); M=2835.187 Da) form, respectively. Oxidation with oxygen leads to an increase of the molecular mass by 15.99 Da and is well known to take place at methionine and tryptophan residues, respectively.

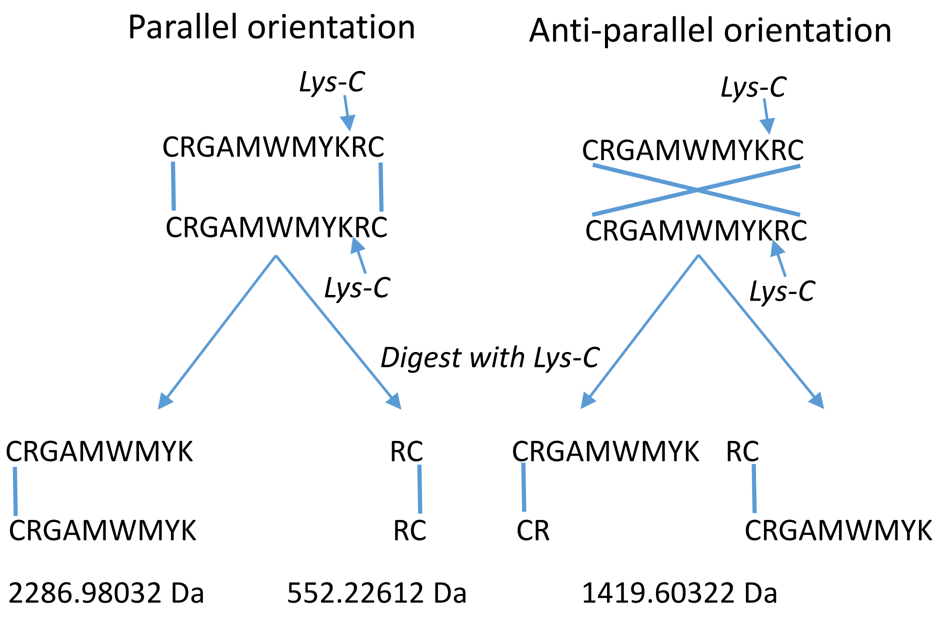

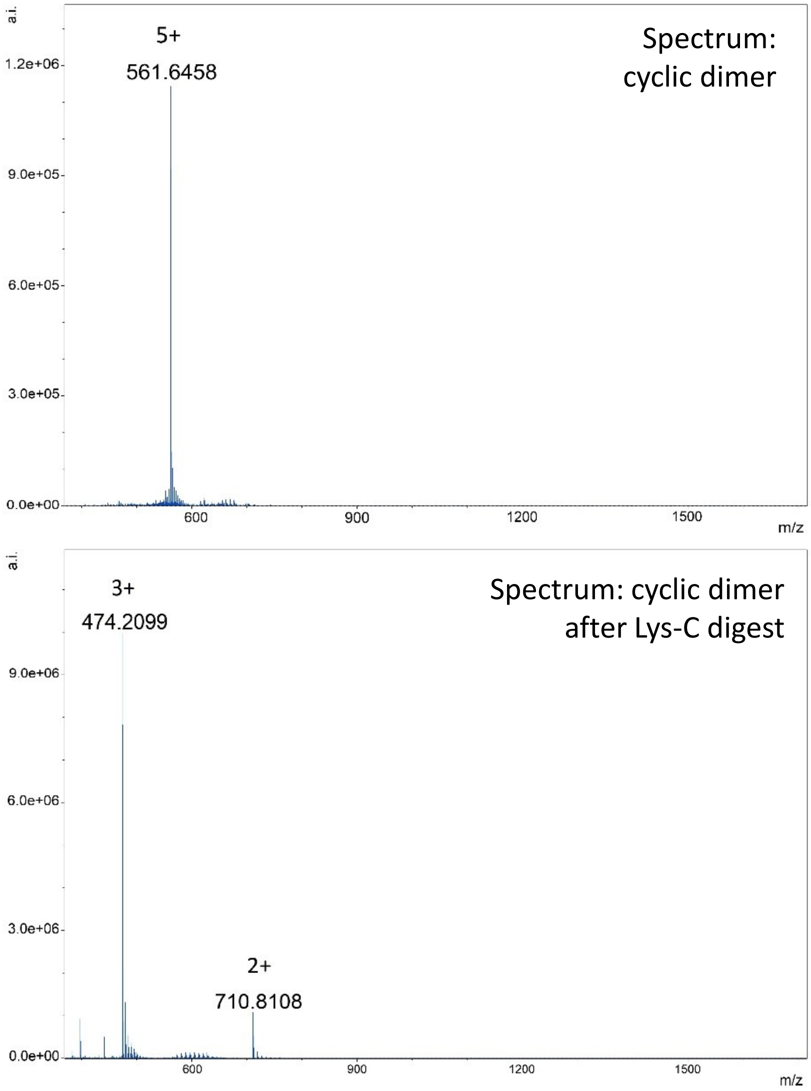

Fig S2. Orientation of purified cyclic dimer: Purified peptide was subjected to Lys-C digestion and analyzed by mass spectrometry. The monoisotopic mass of the undigested peptide (2803.189 Da) as well as of the proteolytic product (1419.605 Da) match the mass values expected for the anti-parallel cyclic dimer.

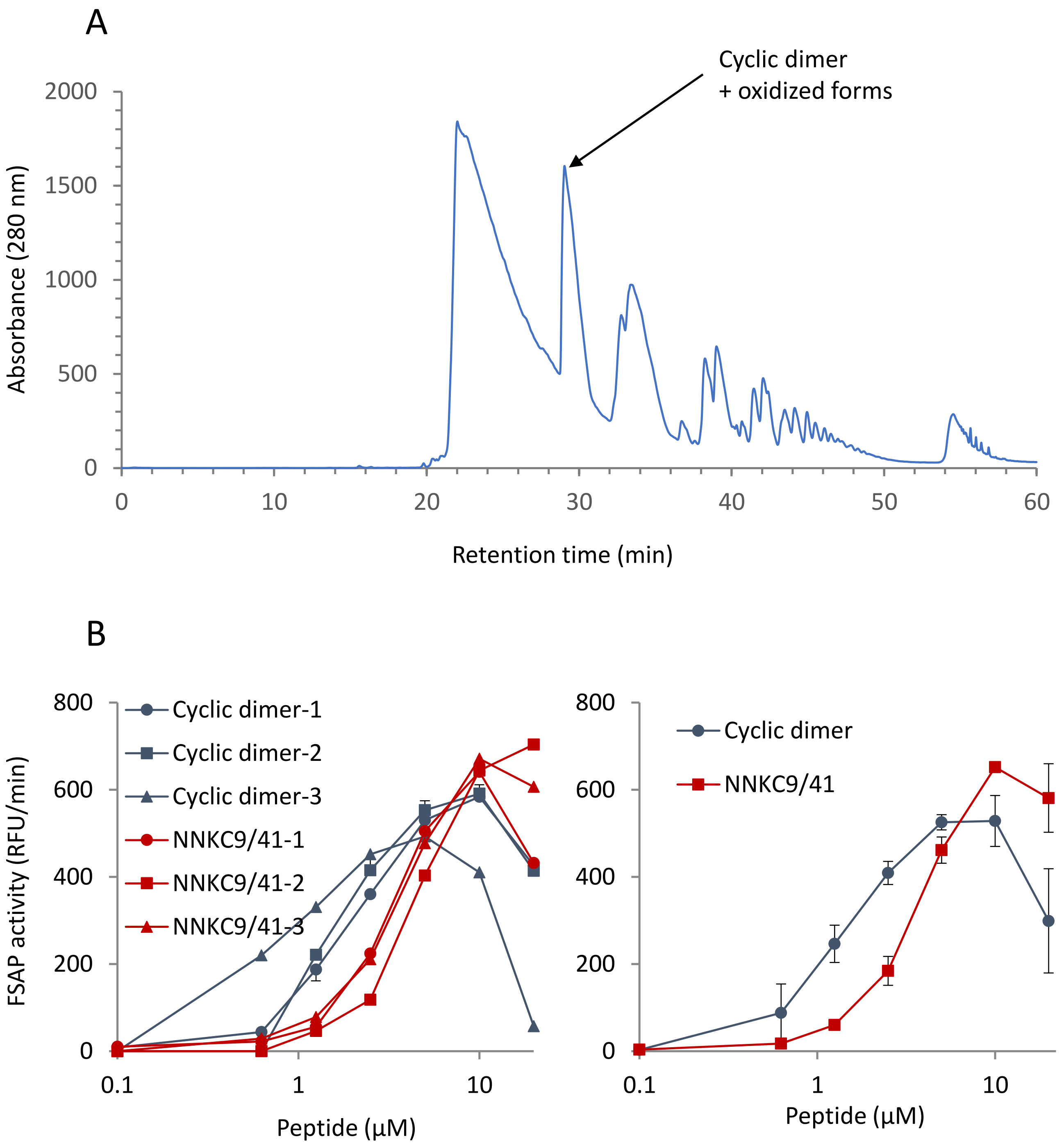

Fig S3. Comparison of purified cyclic dimer and NNKC9/41 on pro-FSAP activation in human plasma: A. Purification of cyclic dimer by HPLC (80% purity). **B.** Hirudin plasma was stimulated with increasing concentrations of cyclic dimer (blue) or NNKC9/41 (red). Turnover of the FSAP fluorescent substrate (Ac-Pro-*D*Tyr-Lys-Arg-AMC) was measured in duplicate (RFU/min, mean). The left panel shows results of experiments on 3 different plasmas (1-3) and the right panel shows mean +/- SEM (n=3 experiments).

Supplementary Fig. S4

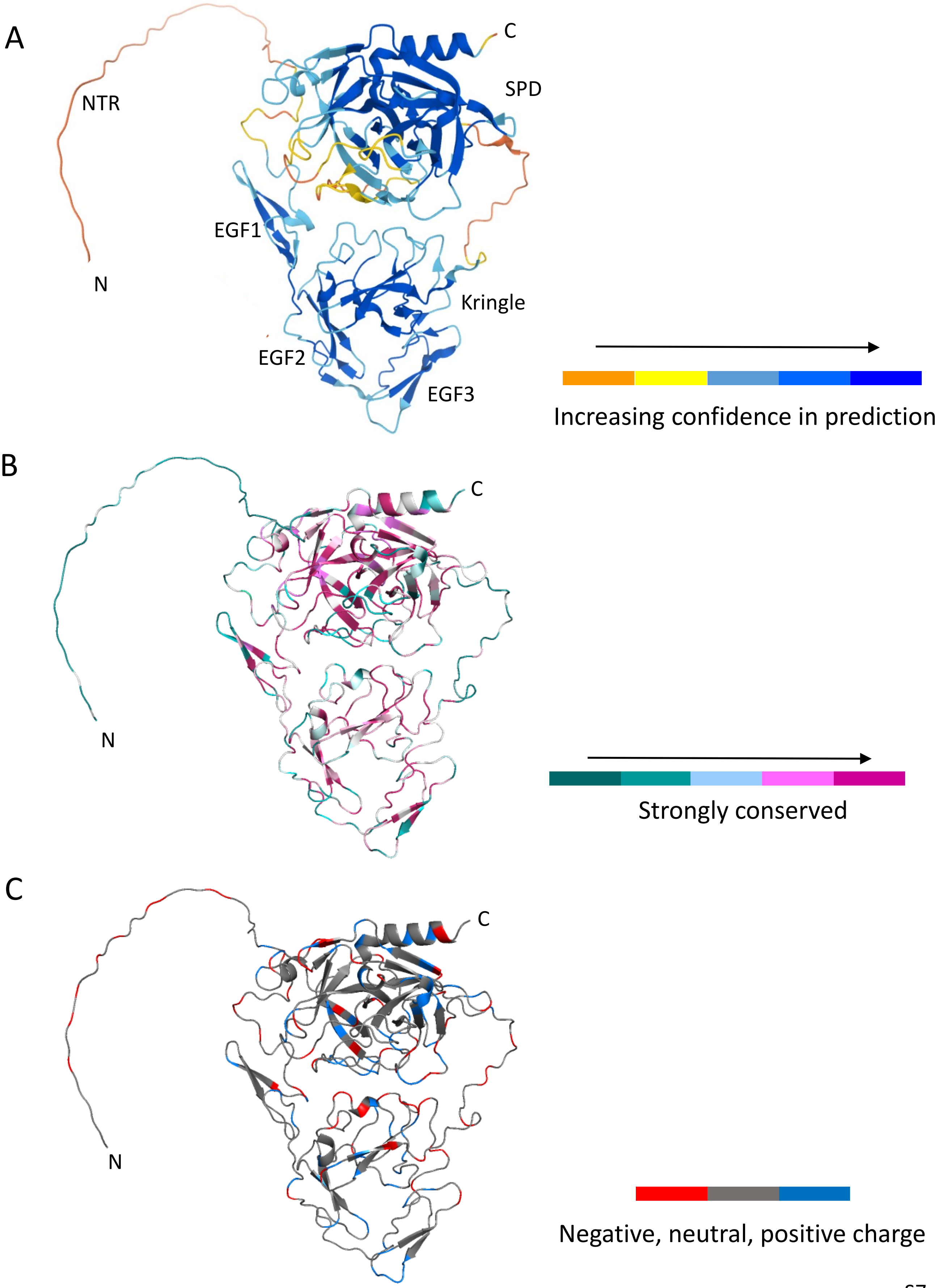

D

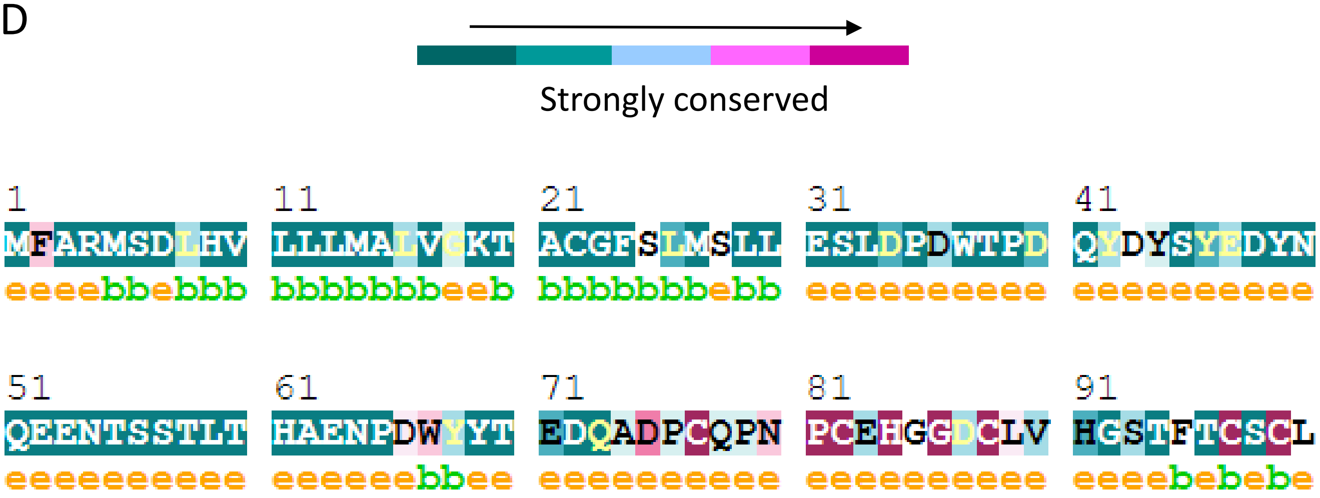

Fig S4. Analysis of FSAP structure and sequence: A. The Alphafold model of human FSAP was colour coded according to the certainty of structure prediction. Low structure prediction (orange) is characteristic of intrinsically disordered domains. B. Alignment of FSAP amino acid sequence from 82 different species from the subphylum vertebrata was performed and each residue was colour coded according to its conservation (dark purple represents 100% conservation). C. The charged residues were coloured (red= negative and blue= positive) in the Alphafold model of FSAP. D. Details of the human sequence in the NTR region (24-72) and its conservation across all species (e = exposed and b = buried).

Supplementary Fig. S5

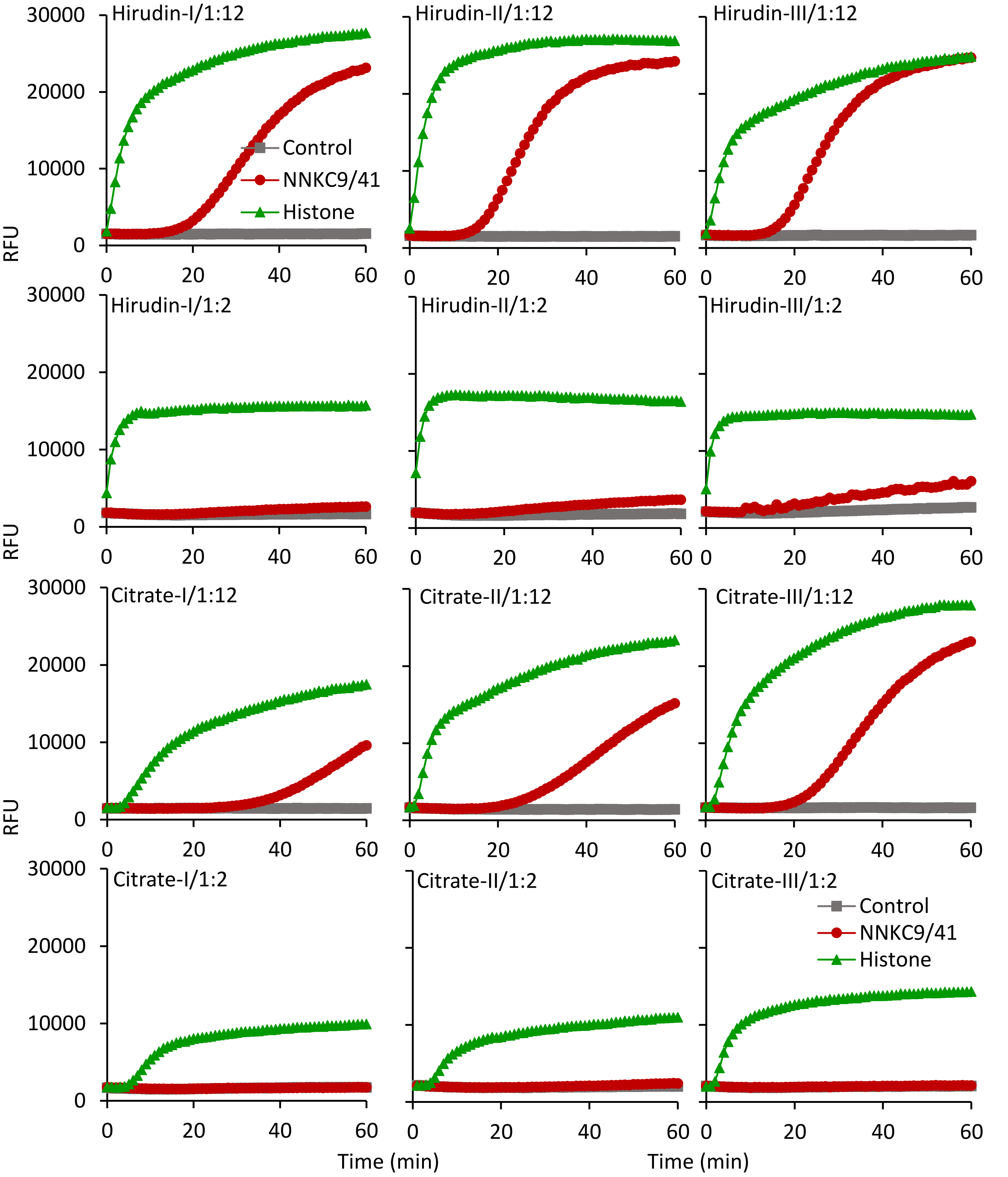

Fig S5. Comparison of hirudin and citrate plasma at different dilutions: Hirudin and citrate plasma (diluted 1:12 or 1:2) from 3 different donors each, was stimulated with NNKC9/41 (10 µM) (red circles) or histones (10 µg/ml) (green triangles). Turnover of the FSAP fluorescent substrate (Ac-Pro-*D*Tyr-Lys-Arg-AMC) was measured (RFU) in duplicate.

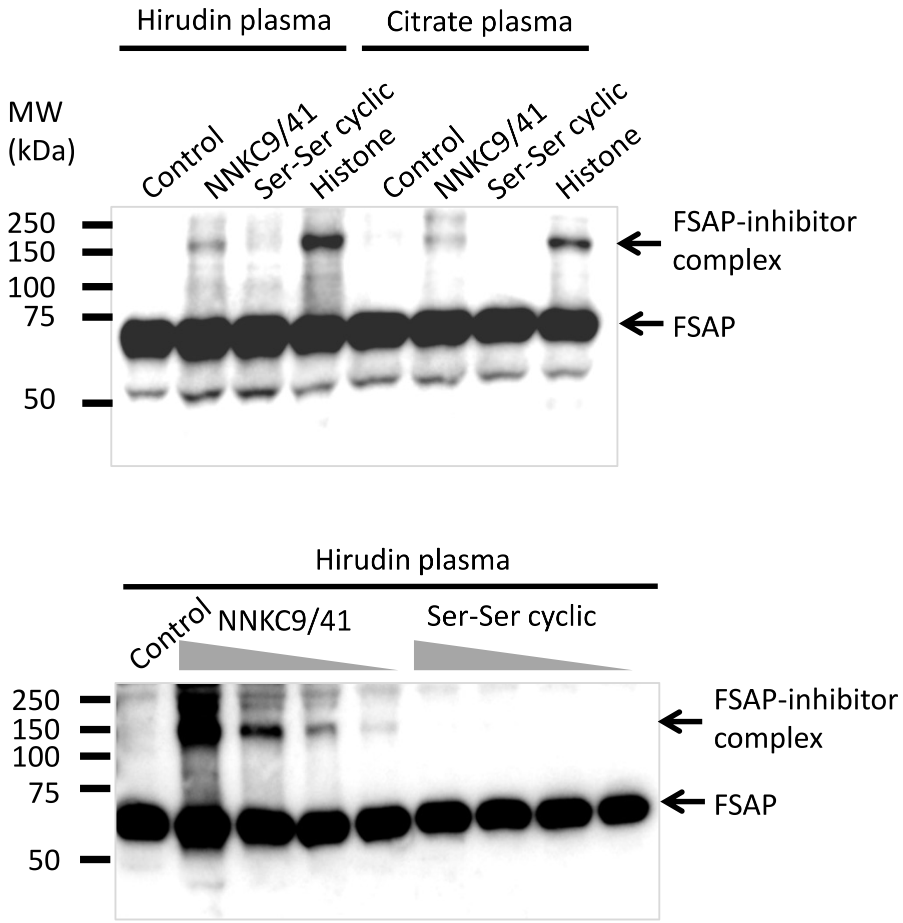

Fig S6. Western blotting of activated plasma: Hirudin or citrate plasma (1:2) dilution was incubated for 1 h at 37 ºC with test substances and the samples were analyzed by Western blotting with an anti-FSAP polyclonal antibody under non-reducing conditions. Top panel: NNKC9/41 and Ser-Ser cyclic peptides (25 µM) were compared to histones (50 µg/ml). Lower panel: 100, 50, 25 and 12.5 µM NNKC9/41 and Ser-Ser cyclic peptide were compared. Arrows on the right indicate the presence of FSAP and FSAP-inhibitor complexes and MW markers are indicated on the left. Results are representative of 3 independent experiments.

Supplementary Fig. S7

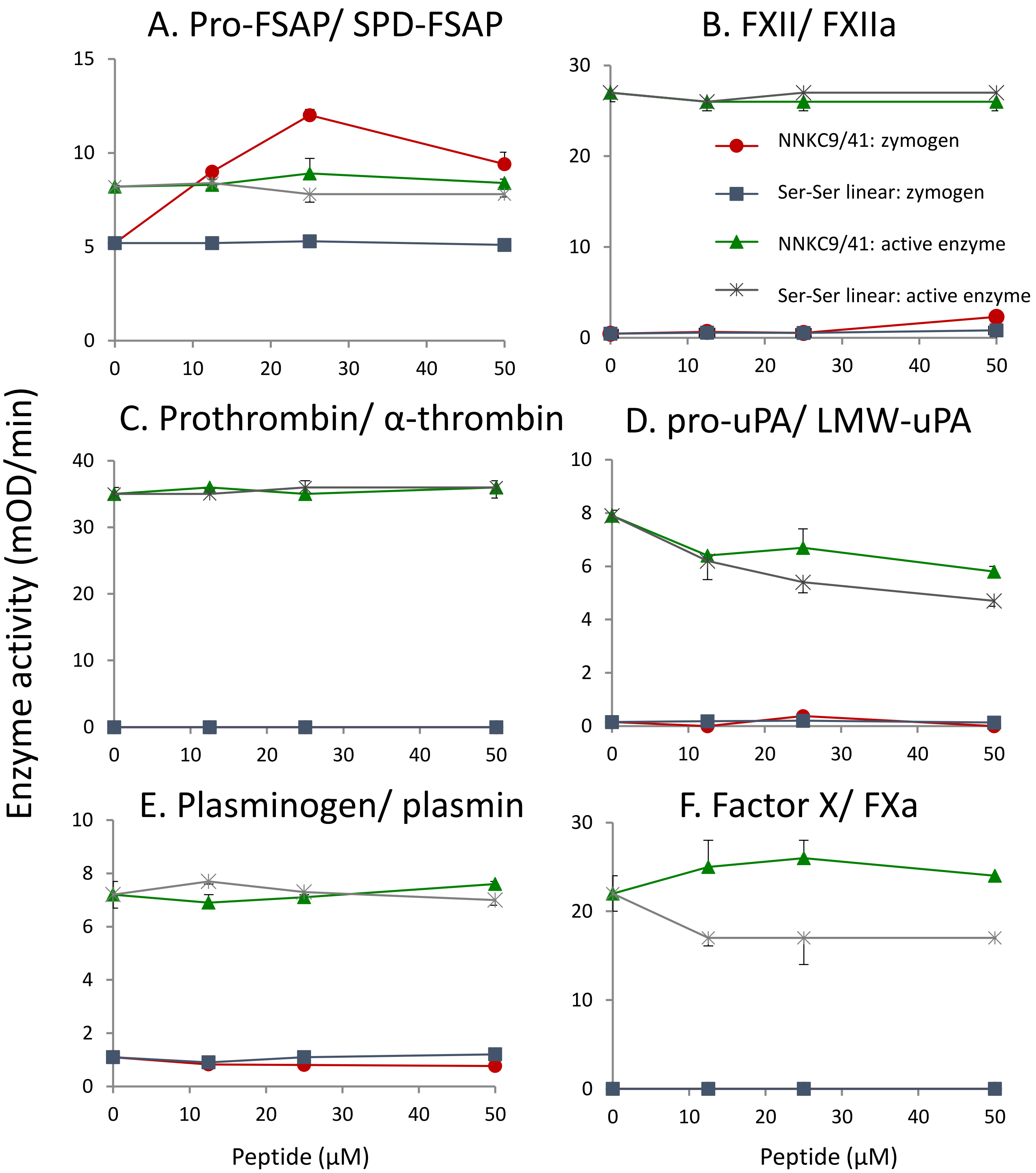

Fig S7. Specificity of NNKC9/41 towards other pro-enzymes: Zymogens and their respective active enzymes were incubated with NNKC9/41 or Ser-Ser linear peptide (0-50 µM) and the enzyme activity was monitored using respective chromogenic substrates. Activity of Factor XII (100 nM) and Factor XIIa (20 nM) was measured with the substrate CS31 (1000 µM). Pro-thrombin (100 nM) and thrombin (20 nM) with CS01 (1000 µM). Plasminogen (50 nM) and plasmin (20 nM) with CS41 (1000 µM). Pro-uPA (100 nM) and uPA (20 nM) with PNAPEP 1344 (250 µM). Factor X (50 nM) and Factor Xa (20 nM) with S-2765 (250 µM). pro-FSAP (20 nM) and recombinant serine protease domain (rSPD) (20 nM) with S2288 (250 µM). All pro-enzymes and enzyme were obtained from Enzyme Research Laboratories (South Bend, IN, USA) except plasminogen and FSAP, which were isolated in house; and pro-uPA was from Grünenthal (Stolberg, Germany). All chromogenic substrates were from Hyphen Biomed (Neuville Sur Oise, France). Experiments were carried out in TBS with Tween-20 (0.1 % wt/ vol) BSA (0.3% wt/vol) and $CaCl_2$ (2 mM). Absorbance at 405 nm was measured over time and the enzyme activity is depicted was measured in duplicates (mean + or - range) and similar results were obtained in 3 independent experiments.

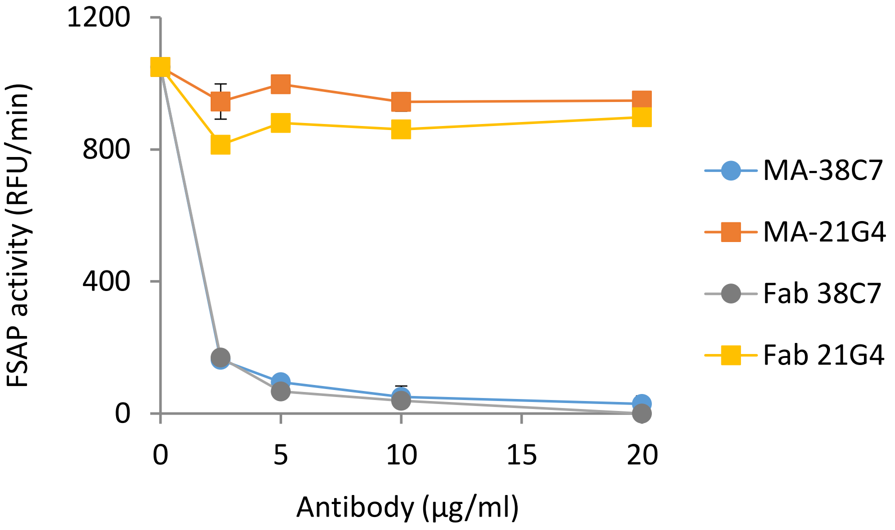

Fig S8. Effect of anti-FSAP antibodies on histone-mediated pro-FSAP activation in plasma: Hirudin plasma was stimulated with histone (25 µg/ml) in the presence of increasing concentration of MA-FSAP-38C7 and a control antibody (MA-FSAP-21G4) or their Fab fragments as indicated. Turnover of the FSAP fluorescent substrate (Ac-Pro-*D*Tyr-Lys-Arg-AMC) was measured in duplicate (mean +/- range). The antibody and Fab fragments were compared on a weight basis and not a molar basis. Similar results were obtained in 2 independent experiments.
